# Treatment outcomes regarding the addition of targeted agents in the therapeutic portfolio for stage II-III rectal cancer undergoing neoadjuvant chemoradiation

**DOI:** 10.18632/oncotarget.21762

**Published:** 2017-10-10

**Authors:** Jin-Tung Liang, Tzu-Chun Chen, John Huang, Yung-Ming Jeng, Jason Chia-Hsien Cheng

**Affiliations:** ^1^ Division of Colorectal Surgery, National Taiwan University Hospital and College of Medicine, Taipei, Taiwan; ^2^ Department of Pathology, National Taiwan University Hospital and College of Medicine, Taipei, Taiwan; ^3^ Department of Radiation Oncology, National Taiwan University Hospital and College of Medicine, Taipei, Taiwan

**Keywords:** bevacizumab, cetuximab, CCRT, rectal cancer, total mesorectal excision

## Abstract

**Background:**

To evaluate the impact of targeted agents in stage II-III rectal cancer undergoing neoadjuvant concurrent chemoradiation therapy (CCRT).

**Method:**

A retrospective study was performed in 124 consecutive patients with clinically T_3_N_0-2_M_0_-staged rectal cancer incorporating targeted agents in CCRT.

**Results:**

Pathologic complete response was detected in 34.2% (n=26) of bevacizumab+FOLFOX-treated patients (n=76), which was significantly higher (p=0.019, post-hoc statistical power =35.87%) than that (n=10, 20.8%) of the cetuximab+FOLFOX-treated patients (n=48). Patients receiving cetuximab+FOLFOX therapy tended to develop severe liver toxicity (91.7%, n=44 versus 17.1%, n=13, p<0.0001), as evaluated by morphologic grading of hepatic steatosis and sinusoidal dilatation in laparoscopy. In the 57 patients with morphologically severe liver toxicity, 36 (63.2%) retained a normal liver function; for the remaining 21 patients with an abnormal liver function, the abnormality was self-limited in 19 patients, whereas 2 cetuximab–treated patients progressed to hepatic failure and mortality. A subset analysis within bevacizumab+FOLFOX-treated patients with either wild-type (n=36) or mutant (n=40) K-ras status indicated K-ras status did not significantly influence the treatment outcomes.

**Conclusions:**

The addition of bevacizumab instead of cetuximab to FOLFOX in the neoadjuvant settings for T_3_N_0-2_M_0_-staged rectal cancer could induce a promising rate of pathologic complete response and lesser hepatotoxicity.

## INTRODUCTION

Preoperative concurrent chemoradiation therapy (CCRT) followed by a standardized total mesorectal excision (TME) is emerging as the new treatment paradigm for advanced low rectal cancer [1–2]. It is generally accepted that the pathologic stage, i.e., the response of cancer to CCRT, is the most powerful predictor for the survival of patients with advanced rectal cancer after CCRT [3–6]. To enhance the tumor response to CCRT, the chemotherapeutic agents in the CCRT protocol have been evolving. Before the year 2000, we routinely used the Mayo regimens as chemotherapy protocol in the CCRT setting. However, since 2003, we have widely adopted FOLFOX (oxaliplatin/5-fluorouracil/leucovorin) as the chemotherapeutic regimen for patients with stage II or III rectal cancer requiring preoperative CCRT, and such regimen can significantly promote the tumor response as reported elsewhere [7–11].

Recently, combining targeted agents (bevacizumab or cetuximab) with chemotherapeutic regimens (XELOX: capecitabine / oxaliplatin, FOLFOX4, and FOLFIRI: 5-fluorouracil / leucovorin / irinotecan), with the high response rate, is increasingly being recognized as state-of-the-art in the neoadjuvant treatment of liver metastasis from colorectal cancer [12–13], although the accompanying liver toxicities such as sinusoidal dilatation and steatosis may paradoxically affect the surgical morbidity of liver resection. Remarkably, since March 2008, the K-ras mutational status of the tumor has been recognized as a biomarker to predict the response to cetuximab therapy [14]. However, the application of cetuximab to the preoperative CCRT regimens has only been sporadically reported to be feasible [15–16]. Therefore, it deserves further investigation whether the safety and efficacy of cetuximab therapy in the neoadjuvant setting of liver metastasis can be extrapolated to that of locally advanced rectal cancer [17], especially in the era in which the K-ras status is reckoned as a useful biomarker.

On the other hand, Willet et al. has provided direct evidence that bevacizumab has antivascular effects in human rectal cancer, and complete pathologic response to bevacizumab and chemoradiation in patients with advanced rectal cancer has been observed thereafter [18–19]. Theoretically, with the unique pharmaceutical features: blockade of angiogenesis, and improvement of the delivery of cytotoxic chemotherapeutic agents to tumor bulk by altering or normalizing the tumor vasculature, bevacizumab may be promising in the clinical context of combination therapy. However, the widespread acceptance of bevacizumab in the neoadjuvant clinical setting is limited by concerns about the bevacizumab-related surgical complications such as bleeding tendency, delayed wound healing, enterocutaneous fistula, and thromboembolism [20].

In the present study, we made a retrospective study to better clarify the impact regarding the addition of bevacizumab versus cetuximab to FOLFOX as preoperative CCRT regimens in patients with stage II-III rectal cancer.

## RESULTS

### Patient accrual and treatment course

124 patients undergoing the treatment program were recruited, with 76 patients categorized to the bevacizumab+FOLFOX group and the other 48 to the cetuximab+FOLFOX group. All patients completed the entire radiotherapy as prescribed without interruptions. However, only 88.7% (n=110) patients complete the whole 6 courses of chemotherapy, with the remaining 14 patients whose chemotherapy were quitted after the second course (n=3), the third course (n=4), the fourth course (n=2), and the fifth course (n=5), respectively.

### Clinicopathologic features of patients before and after CCRT

There was no significant difference between the 2 groups regarding the demographics and various clinicopathologic parameters (Table 1). After CCRT, pathologic complete response (tumor regression grade 5) was detected in 34.2% (n=26) of patients in the bevacizumab+FOLFOX group, which was significantly higher (p=0.019) than 20.8% (n=10) in patients in the cetuximab+FOLFOX group (Table 2), with post-hoc statistical power of 35.87%. However, there was no significant difference between the two groups (75.0%, n=57 in the bevacizumab+FOLFOX group versus 66.7%, n=32, in the cetuximab+FOLFOX group, p>0.05), when the patients with tumor regression grade 5 and 4 (residual tumor cells represented less than 10% in histology) were lumped together and compared.

**Table 1:** Demographics and disease characteristics

Characteristics	Bevacizumab+FOLFOX K-ras		Cetuximab+ FOLFOX K-ras	P-value
	mutant	wild-type	wild-type	
	(n=40)	(n=36)	(n=48)	
**Age (year)**				
Median	57	58	59	NS
Range	28-75	34-75	30-75	
**Gender**				
Male	24	20	26	NS
Female	16	16	22	
**Clinical staging (Pre-CCRT)**				
T3N0	24	20	29	
T3N1	10	9	14	NS
T3N2	6	7	5	
**Differentiation**				
Well	6	5	6	NS
Moderate	32	28	38	
Poor	2	3	4	
**Mucin production**				
+	6	7	7	NS
-	34	29	41	
**L/N metastasis (post-CCRT)**				
+	15	16	20	NS
-	25	20	28	
**Lymphatic/vascular invasion (post-CCRT)**				
+	14	15	17	NS
-	26	21	31	
**Harvested L/N number**				
median	10	11	9	NS
range	4-28	6-24	5-22	
**CRM**				
+	1	1	2	NS
-	39	35	46	

Abbreviation: CRM: circumferential resection margin; L/N: lymph node; NS: not statistically significant.

**Table 2:** Histopathologic features of the primary colorectal cancer in response to CCRT

Characteristics	Bevacizumab+FOLFOX K-ras		total	Cetuximab+FOLFOX K-ras	P-value^*^
	mutant	wild-type		wild-type	
	(n=40)	(n=36)		(n=48)	
**Pathologic response**					
Grade 0: no regression distant metastasis (progressive disease)	1	1	2	2	
Grade 1: dominant tumor mass (>50%) with obvious fibrosis (no response)	3	4	7	4	
Grade 2: obvious tumor cells (25-50%) with dominant fibrosis (stable disease)	2	3	5	6	
Grade 3: few tumor cells (10-25%) with dominant fibrosis (partial response)	3	2	5	4	
Grade 4: very few tumor cells (<10%) in fibrotic tissue (partial response)	17	14	31	22	
Grade 5: no tumor cells, only fibrotic mass or acelluar mucin pool (complete response)	14	12	26 (34.2%)	10 (20.8%)	P=0.019
**Concomitant histologic changes**					
Vessel intima fibrosis	28			36	NS
Foreign body reaction	16			20	NS
Mucin pooling	24			25	NS
Calcification / cholesterol cleft	15			22	NS
Ulceration with mucosa regeneration	32			40	NS

^*^The p-value was calculated based on the comparison between total case number of bevacizumab-treated and cetuximab-treated patients

When the pathologic complete response rate was compared among T_3_N_0_, T_3_N_1_, and T_3_N_2_ groups of patients (Table 1), we found that it had been very difficult to achieve a complete remission of cancer in metastasized lymph nodes, because it was only detected in 11.8% (n=6/51) of patients with clinically positive lymph node metastasis.

### Adverse effects of CCRT and surgical complications

**Figure 1:**
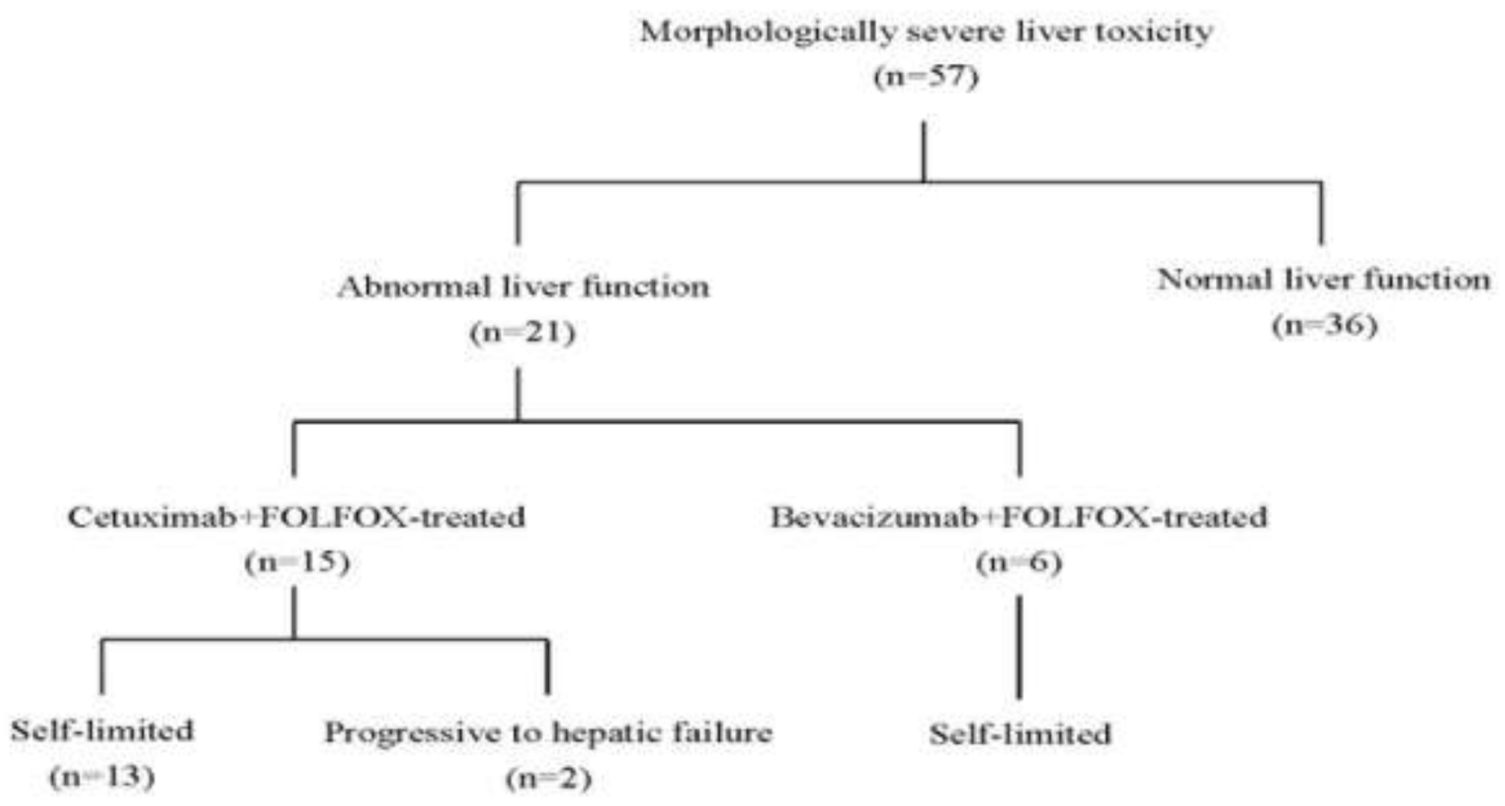
Profile of liver injury in patients receiving preoperative CCRT

There was no difference between the bevacizumab+FOLFOX and cetuximab+FOLFOX group of patients in regard to CCRT-related severe adverse events (9.2%, n=7 versus 8.3%, n=4, Table 3), and surgery-related morbidity (13.2%, n=9 versus 8.3%, n=4) or mortality (1.3%, n=1 versus 4.2%, n=2, Table 4). However, compared with the bevacizumab+FOLFOX therapy, the cetuximab+FOLFOX therapy tended to have less blood loss during surgery (Table 3) but was associated with more severe liver toxicity (91.7%, n=44 versus 17.1%, n=13, p<0.0001) (Table 5). Remarkably, 36 (63.2%) of the patients with morphologically severe liver toxicity (n=57) remained a normal liver function, as evaluated by serum level of alanine aminotransferase (ALT) after CCRT (Figure 1). Even for the 21 patients with abnormal liver function (ALT level: median: 94 U/L, range: 54-244 U/L, n=21), the abnormality was self-limited and recovered within 3 months after surgery in most patients (90.4%, n=19) However, 2 cetuximab–treated patients did progress to hepatic failure and mortality after surgery. The cause of death in these 2 patients was ascribed to hepatic failure because their preoperative function was normal and ultimately developed an impairment of liver function meeting the Child C criteria.

**Table 3:** Adverse effects in patients with rectal cancer undergoing CCRT

Characteristics	Bevacizumab+FOLFOX K-ras		total	Cetuximab+FOLFOX K-ras	P-value^*^
	mutant	wild-type		wild-type	
	(n=40)	(n=36)		(n=48)	
**FOLFOX-related toxicity**					
Neutropenic fever	1	1	2	2	NS
Severe diarrhea	0	0	0	1	NS
Neuropathy					
Grade 1	19	14	33	14	NS
Grade 2	21	22	43	26	NS
**Bevacizumab-spcific adverse effects**					P<0.001
UGI Bleeding	2	1	3	1	
Hypertension	2	3	5	1	
Proteinuria	1	1	2	1	
Arterial / venous thromboembolic events	1	1	2	0	
Wound-healing complications	2	1	3	1	
Gastrointestinal perforation	1	1	2	0	
**Cetuximab-related adverse effects**					P<0.001
Acne/ Acneiform rash					
Grade 1	3	2	5	20	
Grade 2	1	1	2	28	
Hand-foot skin reaction (Paronychia)					
Grade 1	1	2	3	30	
Grade 2	2	2	4	18	
**Discontinuations due to a severe adverse event** (neutropenic fever, severe diarrhea, UGI bleeding, GI perforation, thromboembolism)	5	2	7	4	NS

Abbreviation: UGI: upper gastrointestinal tract;

^*^The p-value was calculated based on the comparison between total case number of bevacizumab-treated and cetuximab-treated patients

**Table 4:** Surgical complications in patients with rectal cancer after CCRT

Characteristics	Bevacizumab+FOLFOX K-ras		total	Cetuximab+FOLFOX K-ras	P-value^*^
	mutant	wild-type		wild-type	
	(n=40)	(n=36)		(n=48)	
**ASA grade**					
I	21	14		26	
II	17	20		19	P<0.0001
III	2	2		3	
**Types of operation**					
APR	4	4		5	NS
LAR	31	28		39	
Pull-through	5	4		4	
**Surgical morbidity**					
Blood loss (ml, median/range)	340 (100-800)	350 (100-1000)	450 (100-1000)	130 (40-600)	P<0.0001
Deep vein thrombosis	1	1	2	1	
Pelvic abscess	1	1	2	1	
Enterocutaneous fistula	1	1	2	1	
Perineal fistula	1	0	1	1	
Rectovaginal fistula	1	1	2	0	
**Surgical mortality**					
Hepatic failure	0	0	0	2	
AMI	1	0	1	0	

^*^Abbreviation: APR: abdominoperineal resection; LAR: low anterior resection; Pull-through: abdominoanal pull-through procedure with coloanal anastomosis; ^*^ASA: American society of anesthesiology; NS: not statistically significant; AMI: acute myocardial infarction

^*^The p-value was calculated based on the comparison between total case number of bevacizumab-treated and cetuximab-treated patients

**Table 5:** The concomitant liver toxicity in response to CCRT for rectal caner

Characteristics	Bevacizumab+FOLFOX K-ras		total	Cetuximab+FOLFOX K-ras	P-value^*^
	mutant	wild-type		wild-type	
	(n=40)	(n=36)		(n=48)	
**Liver toxicity**					
**Overall**					
Severe	8	5	13(17.1%)	44(91.7%)	P<0.0001
Moderate	20	17		4	
Mild	12	14		0	
**Sinusoidal dilatation (blue liver)**					
Severe	6	3		25	
Moderate	14	13		23	
Mild / absent	20	20		0	
**Liver steatosis (yellow liver)**					
Severe	2	2		5	
Moderate	14	11		30	
Mild / absent	24	23		13	

^*^The p-value was calculated based on the comparison between total case number of bevacizumab-treated and cetuximab-treated patients

### Survival benefits

**Figure 2:**
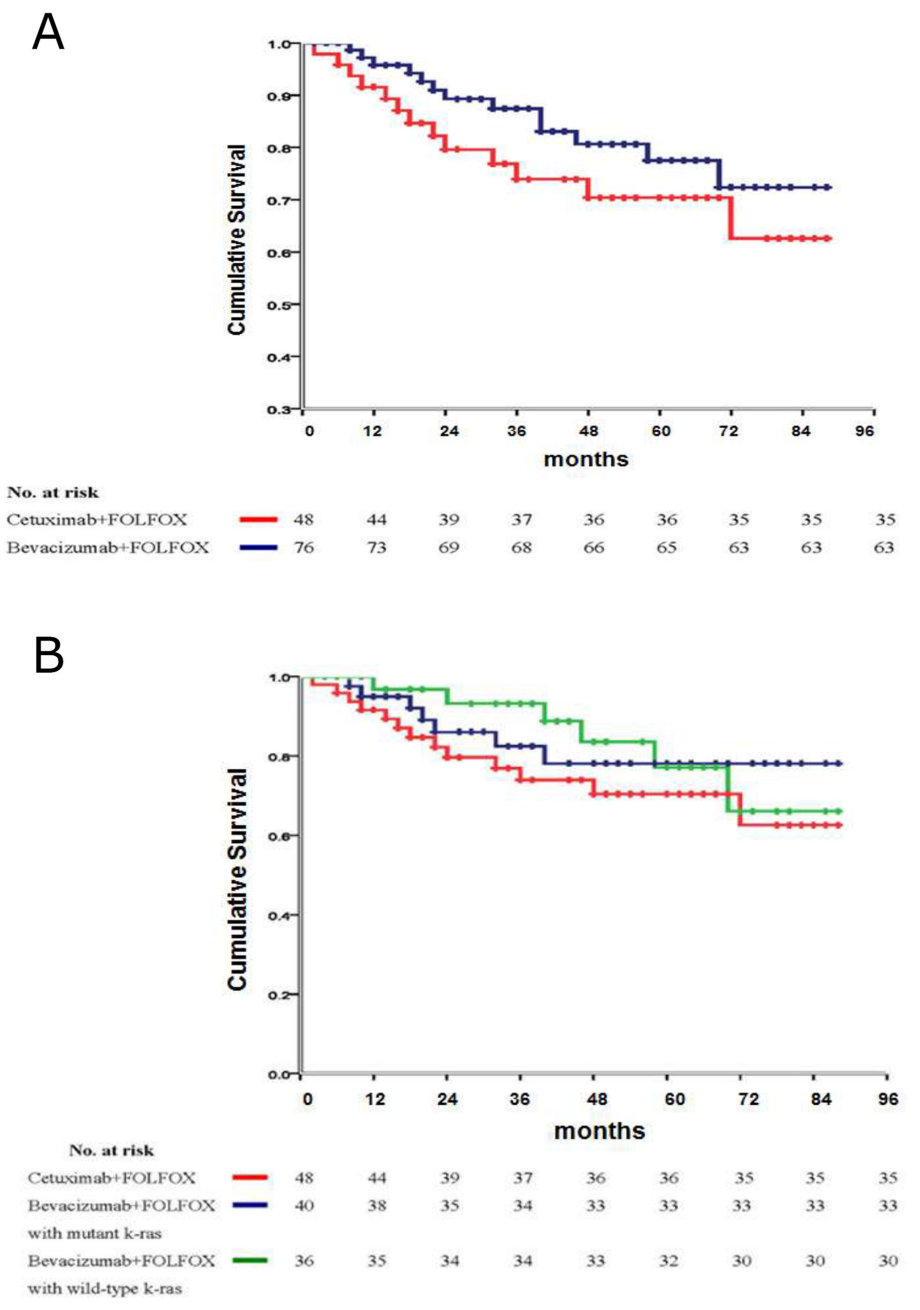
**(A)** Disease-free survival of patients receiving either bevacizumab or cetuximab in the CCRT treatment protocol. **(B)** Subset survival analysis regarding the influence of k-ras status on disease-free survival in bevacizumab- treated patients, in comparison with cetuximab- treated patients.

Kaplan-Meier analysis of long-term disease-free survival (follow-up time: median: 82 months, range: 63-94 months) and recurrence pattern shows that the oncologic efficacy of bevacizumab-treated patients was better than that of cetuximab-treated patients (p=0.0207) (Figure 2-A, Table 6). A subset analysis within bevacizumab-treated patients indicated that the k-ras status was not associated with any clinicopathologic features (Table 1) and treatment outcomes (Table 2-5), as was shown (p=0.857) in disease-free survival (Figure 2-B).

**Table 6:** Comparison of recurrence patterns between bevacizumab+FOLFOX (n=76) and cetuximab+FOLFOX (n=48) treated patients

recurrence patterns	Bevacizumab+FOLFOX K-ras		total	Cetuximab+FOLFOX K-ras	P-value
	mutant	wild-type		wild-type	
	(n=40)	(n=36)		(n=48)	
**Distant metastasis**					
Liver	1	1	2	0	
Lung	0	1	1	1	
Bone	0	0	0	1	
Para-aortic lymph nodes	1	0	1	0	
Peritoneal carcinomatosis	0	1	1	0	
Multiple organ metastases					
Liver + Lung	1	1	2	1	
Liver + Bone	0	0	0	1	
**Local recurrence**					
Presacrum	0	1	1	1	
Pelvic side wall	1	0	1	1	
**Distant metastasis plusLocal recurrence**					
Lung + perineal wound	1	0	1	1	
Lung + Bone + Presacrum	0	1	1	1	
Bone + Pelvic side wall	1	0	1	1	
Liver + Presacrum	0	0	0	1	
Liver + Pelvic side wall	1	0	1	1	
**Total: n (%)**					
Locoregional recurrence	4	2	6 (7.9%)	7 (15.2%)	P=0.2467
Distant metastasis	6	5	11(14.5%)	9 (19.6%)	P=0.6182

## DISCUSSION

The present retrospective study has shown that bevacizumab+FOLFOX can achieve a pathologic complete response rate of clinical stage II-III rectal cancer of up to 34.2%, which is much higher than that of any other contemporary reported case series [8–11, 28]. Remarkably, when combined with radiotherapy, both oxaliplatin and irinotecan have been reported to be promising. Irinotecan (a topoisomerase I inhibitor) and oxalipaltin (causing intrastand cross-links in DNA), are DNA damaging agents, and theoretically both of them can be effective as a radiosensitizer. However, the Taiwan National Insurance Bureau only approves FOLOFOX regimen in the adjuvant setting of stage III rectal cancer, based on the evidence from Mosaic clinical trial [7], in which FOLFOX was beneficial in the adjuvant setting of stage III colon cancer. Moreover, we are concerned about the adverse effect of severe diarrhea superimposed by irinotecan on radiotherapy. Therefore, in this study, only an oxaliplatin-based regimen was devised and implemented. Previous reports have indicated that FOLFOX regimen can induce the complete response rate of rectal cancer to CCRT of up to 25% [7–11]. We speculated that the even higher complete response rate in the present series was attributed to the synergistic effect of bevacizumab to the FOLFOX regimen. Simultaneously, the present study again indicated that the surgical complications inherent to the use of bevacizumab such as bleeding tendency, thromboembolism, and enterocutaneous fistula did occur, but their incidence was low provided that the surgical intervention was postponed to 4-6 weeks after the final dose of bevacizumab therapy.

There have been ample evidences to prove that a higher complete response rate of rectal cancer to CCRT can translate into better local control and improved survival of patients [3–6]. In the present case series, only 2 (7.7%) of the 26 patients with complete response to bevacizumab+FOLOFOX therapy developed distant metastasis, in contrast to 11 (22.0%) patients who developed distant metastasis with or without simultaneous local recurrence in the remaining 50 patients without complete response (Table 2, 6). Therefore, we think that the encouraging oncologic complete response rate of advanced rectal cancer to bevacizumab+FOLFOX regimens in the present retrospective study may be a reasonable surrogate for the final oncologic results of the other ongoing further studies.

It needs to be mentioned that the criteria for pathologic staging of rectal cancer after CCRT, in which tumor regression grade (TRG) is used (Table 2), is quite different from that for rectal cancer without CCRT, in which the traditional TNM (tumor-node-metastasis) staging system is used. In this study, we frequently observed a rectal cancer after CCRT was with a high TRG (residual tumor amount less than 10%) but remained a T3 stage in traditional TNM staging system, just because the scanty residual cancer cells were still accumulated in the subserosa layer of the recta wall, as was shown in the 4 cases of positive circumferential resection margin.

The mechanisms for the promising enhancement of the pathologic complete response by bevacizumab have been addressed by some authors [18–19], but are yet unproven. Willet et al. stressed the importance of bevacizumab in the normalization of tumor vasculature, which subsequently reversed the compromised delivery of oxygen and chemotherapeutic agents to the tumor bed and therefore induced better radio-chemotherapeutic sensitivity of the rectal cancer, as it was indirectly evidenced by the increased plasma level of mediators such as soluble vascular endothelial growth factor receptor (sVEGFR1), vascular endothelial growth factor (VEGF), placental-derived growth factor (PIGF), interleukin 6 (IL-6) during treatment, and circulation endothelial cells (CECs) after treatment [28]. A few researchers argument against the oncologic benefits of preoperative CCRT for rectal cancer by showing that better local control of the tumor was not equal to longer overall survival of patients [29, 30]. However, based on the present study, we encourage the inclusion of bevacizumab in the preoperative CCRT treatment protocol because we believe that a higher rate of pathologic complete response can translate into a better local control of the primary cancer, which can therefore minimize the local recurrence rate and thus helping patients obviate the local recurrence-related sufferings such as intractable pelvic pain, genitourinary, and anorectal dysfunction.

It has been reported that liver steatosis was associated with the use of 5-fluorouracil and oxaliplatin through the mechanisms such as dysregulation in the production of lipoprotein and glycogen storage in the liver, and disruption of mitochondria, leading to increased oxidation of cellular proteins [31]; whereas sinusoidal dilatation of the liver has been considered to be uniquely caused by oxaliplatin, which can directly injure the endothelial cells lining the sinusoids of the liver [27–28, 30–32]. Remarkably, in the present study, we found that the FOLFOX plus bevacizumab-treated patients had a lower severity of sinusoidal injury; whereas cetuximab seemed to aggravate the liver toxicity. Although some previous authors suggested that bevacizumab may protect against sinusoidal damage, the underlying biochemical mechanisms remain unclear and further mechanistic studies are still needed [33–34]. On the other hand, we think that the liver toxicity may be aggravated by the effects of antibody-dependent cytotoxicity (ADCC) inherent in the pharmacodynamics of cetuximab. Seeing the acneiform skin reaction and the tendency to develop lymphocyte infiltration in tumor histology in the present case series, we thus speculated that such a systemic inflammatory response aroused by cetuximab may potentiate FOLFOX in causing more severe liver toxicity. However, unlike the condition involving liver resection for the treatment of metastatic colorectal cancer [35], the morphologic liver toxicities associated with preoperative CCRT did not significantly increase the perioperative morbidities for the surgical resection of rectal cancer in the present case series. Usually, patients with severe morphologic liver toxicity induced by the aggressive preoperative CCRT using targeted agents retained a normal liver function. Even in the patients with an abnormal liver function (36.8%, n=21), the alanine aminotransferase (ALT) was only moderately elevated and in most of such patients, abnormality of liver function was self-limited and recovered within three months after surgery. However, two cetuximab-treated patients were progressive to hepatic failure and surgical mortality.

In summary, the present study has shown that, compared with cetuximab, bevacizumab can induced higher pathologic complete response rate for patients with T_3_N_0-2_-staged advanced low rectal cancer requiring preoperative CCRT. Despite pre-operative chemotherapy with FOLFOX/XELOX with or without cetuximab (EXPERT-C trial) [36] has been used to treat locally advanced rectal carcinoma, concomitant to radiation or before chemoradiation, the use of monoclonal antibodies (bevacizumab or cetuximab) is still considered an investigational strategy. Data from the large randomized trial evaluating the role of neoadjuvant chemotherapy without radiation is pending (PROSPECT trial) [37]. What's more, Gasparini et al. reviewed the Phase II studies on fluoropyrimidines and bevacizumab+ RT, and showed that there had been no evidence that oxaliplatin had added significant higher pathologic complete response rate for locally advanced rectal cancer [38]. Therefore, The present retrospective study should mandate further multi-centered randomized prospective clinical trials to establish the oncologic efficacy of bevacizumab in the daily practice of preoperative CCRT for low rectal cancer.

## MATERIALS AND METHODS

### Patient recruitment and stratification

Prospectively enrolled clinicopathologic data of consecutive patients with clinically T_3_N_0-2_M_0_-staged distal rectal cancer and treated between March 2008 and August 2011 at the Colorectal Division, Department of Surgery, National Taiwan University Hospital, were retrospectively reviewed. This study has obtained approval from National Taiwan University Hospital (NTUH) Research Ethics Committee (REC) and was designated as: 201103126MB. Distal rectal cancer was defined as a tumor located within 10 cm above the anal verge. The pretreatment clinical stage of the tumor was determined by the selective use of transrectal ultrasonography (TRUS), magnetic resonance imaging (MRI), multi-slice spiral computed tomography (CT), and/or positron-emission tomography (PET). Patients with abnormal liver function or severe liver steatosis, as screened by preoperative blood sampling and ultrasonography, were excluded. The K-ras status of the cancer was determined by pretreatment colonoscopic tumor biopsy. The codon 12 and 13 of the K-ras gene were screened by a direct sequencing of the polymerase-chain-reaction-enriched products, as reported before [14, 21].

In regard of the indication for treatment arm, the choice of cetuximab or bevacizumab is firstly according to the mutation status of K-ras gene. If K-ras gene is mutant, the patient can only choose the FOLFOX + bevacizumab regimen in CCRT protocol. However, if the K-ras gene is wild-type, generally, the choice of FOLFOX + bavacizumab or FOLFOX + cetuximab is up to the patients’ preference. In such condition, the patients with poor economic status usually chose FOLFOX + bevacizumab because it was cheaper.

The patients were stratified into 3 groups: bevacizumab +FOLFOX-treated and with wild-type k-ras status, bevacizumab+FOLFOX-treated and with mutant k-ras status, and cetuximab+FOLFOX-treated and with wild-type k-ras status. Ample studies regarding head-to-head comparison between bevacizumab and cetuximab have shown that cetuximab is superior to bevacizumab in metastatic colorectal cancer with wild-type K-ras gene. And, some reports showed that the mutational status of K-ras gene is associated with patients’ prognosis. Therefore, the subset analysis is done to clarify the influence of K-ras gene *per se* on the patient survival.

The primary endpoints of the present study were the rate of the pathologic complete response and safety profiles, especially the chemotherapy-induced liver toxicities. We first compared the clinicopathologic features between cetuximab+FOLFOX and bevacizumab+FOLFOX treated patients, and then a subset analysis was made within bevacizumab+FOLFOX treated patients to investigate the influence of K-ras status on the treatment outcomes.

### Treatment

**Figure 3:**
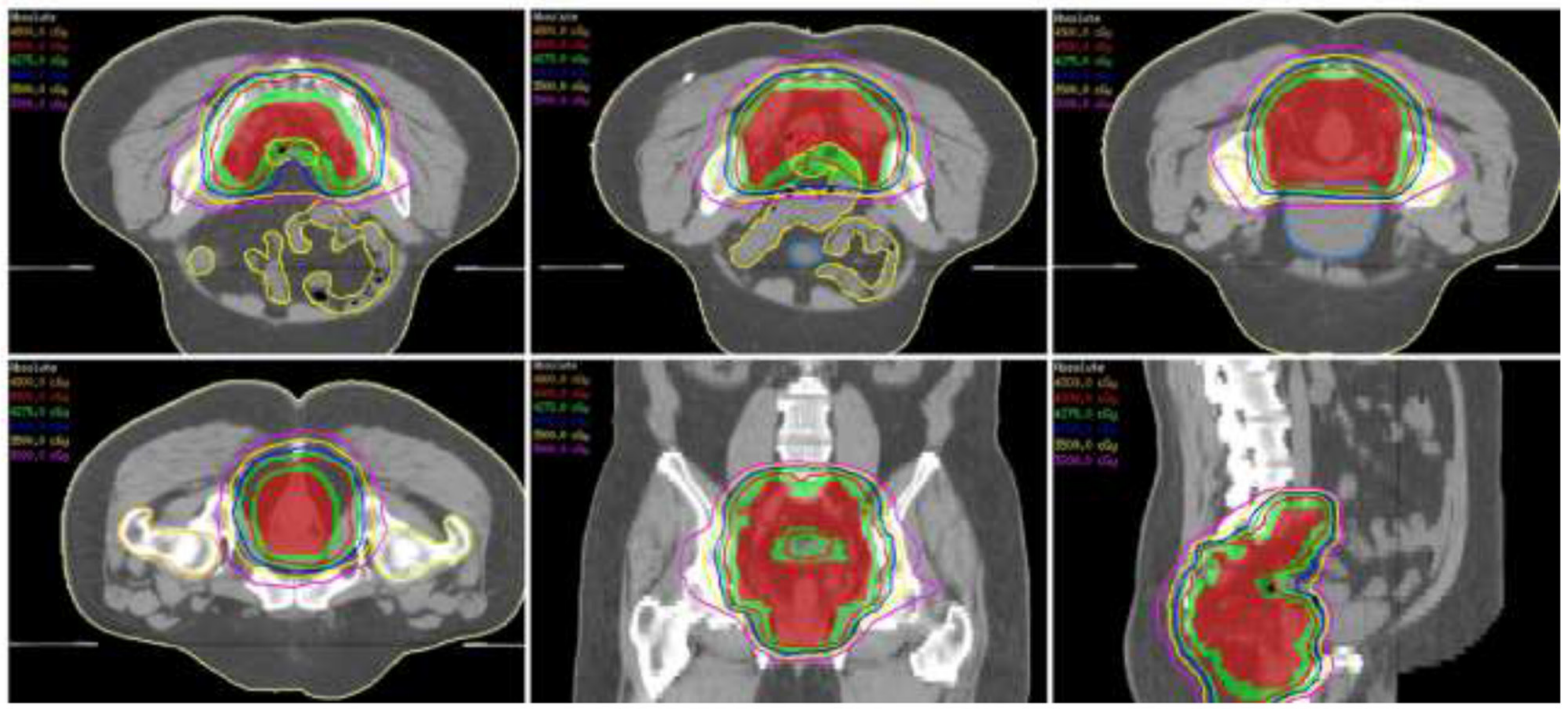
The isodose distributions for one representative patient planned by volumetric modulated arc therapy are shown with axial (upper panel and lower-left), coronal (lower-middle) and sagittal (lower-right) views The red and green colorwash contours represent the clinical and planning target volumes, respectively.

The eligible patients were subjected to the preoperative CCRT regimen, which is shown in Table 7 and Figure 3. Briefly, immediately after the completion of clinical staging, the patients received the first course of chemotherapy, which was followed by long-course radiation. Generally, the interval between the first course of chemotherapy and the initiation of radiation is one week. The patients underwent 6 cycles of chemotherapy at 2-week intervals. The 4500 cGy radiotherapy, 25 fractions in 5 consecutive weeks, was given synchronously in-between the time course of chemotherapy. The CCRT treatment protocol was completed at 14 weeks after the initial diagnosis and re-staging of patients was performed at this time point. Thereafter, a standardized laparoscopic total mesorectal excision (TME), as described in our previous publications [22], was scheduled for the patients, generally 6 weeks after the final dose of chemotherapy (bevacizumab or cetuximab plus FOLFOX).

**Table 7:** Treatment plan and the regimen for radiotherapy and chemotherapy

1. Patients with T_3_N_0-2_ rectal cancer were prospectively recruited and stratified according to K-ras status and chemotherapeutic regimens (FOLFOX + bevacizumab vs. FOLFOX + cetuximab).
2. Pre-CCRT staging of cancer and CRM (circumferential resection margin) estimation were made based on the selective use of Transrectal Ultrasonography (TRUS), MRI (magnetic resonance imaging), multislice spiral CT and/or PET (positron emission tomography)-scan.
3. Radiotherapy (4500 cGy during 5 weeks/25 fractions) starts 2 weeks after the initial diagnosis and the radiotherapy is performed synchronously within the time course of 6-cycle chemotherapy.
4. Chemotherapy FOLFOX (5-FU: 2600 mg/m^2^; leucovorin: 300 mg/ m^2^; maximum 500 mg; oxaliplatin: 85 mg/m^2^) + bevacizumab (5 mg/kg) or cetuximab (450 mg/m^2^), biweekly, 6 cycles, starts immediately after the initial diagnosis.
5. Laparoscopic total mesorectal excision (TME) was done 6 weeks after the final dose of chemotherapy.

### Histopathologic scrutiny of the resected tumor specimen after CCRT

The preparation of tissue specimens for the assessment of tumor response to CCRT and the quality of TME were performed with reference to guidelines from Nagtegaal et al. [23] and the contemporary literature review [24].

The circumferential resection margin (CRM) was considered to be tumor-free when the safety margin was more than 2 mm. The pathological response of rectal cancer to CCRT was graded from 0-5 (Table 2) according to the criteria modified from Dworak et al. [25–26].

### Evaluation of liver toxicity to CCRT

**Figure 4:**
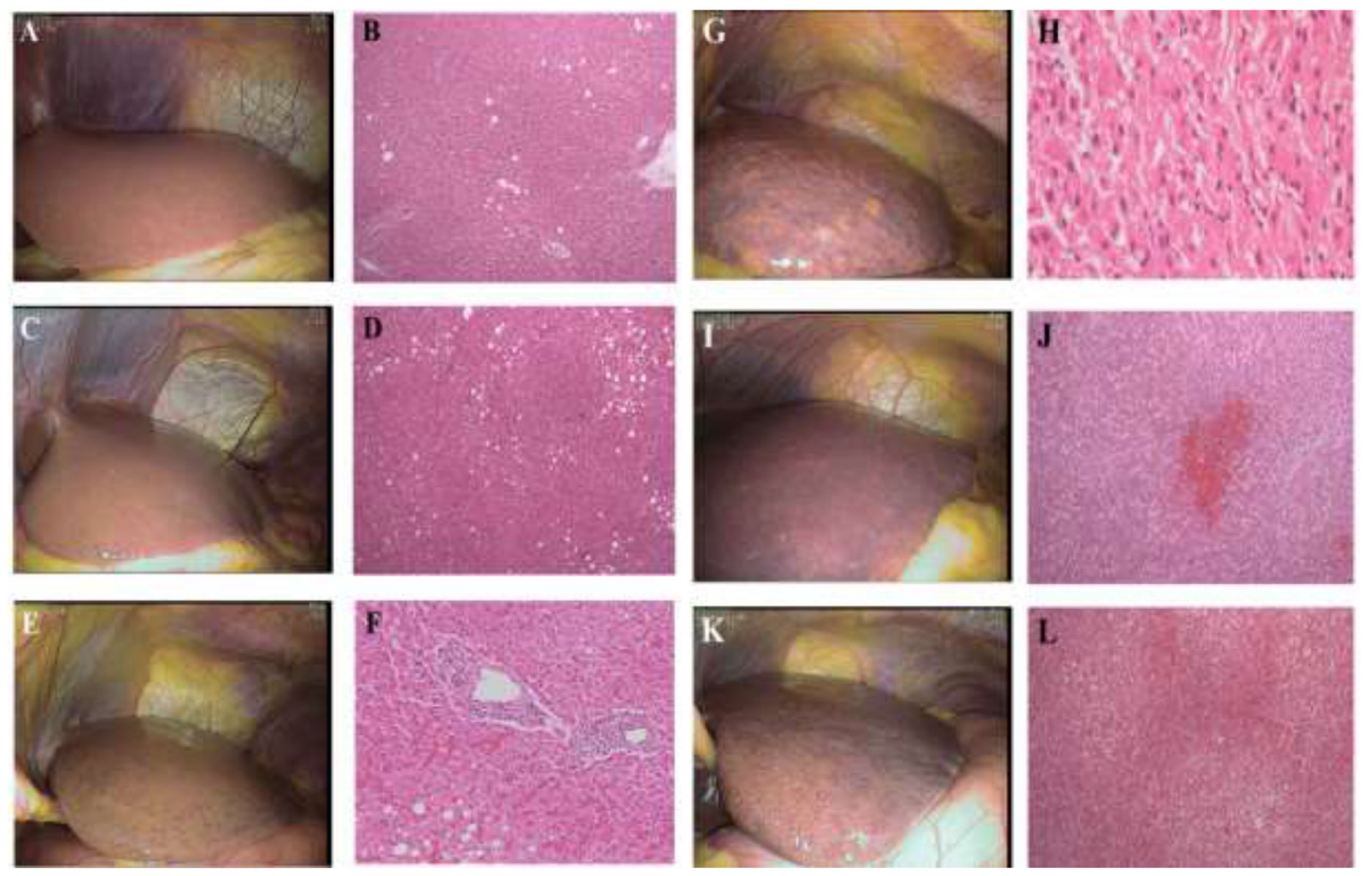
**(A, B)** A case of normal control of the liver toxicity. The steatosis is less than 3% of the histologic view. **(C, D)** A case of mild steatosis without sinusoidal dilatation. The steatosis is less than 33% of the histologic view. **(E, F)** A case of moderate sinusoidal dilatation (33-66%) in the laparoscopic and the histologic view. **(G, H)** A case of severe liver injury with marked sinusoidal dilatation in laparoscopic view and microvesicular steatosis in histology. **(I, J)** A case of severe sinusoidal dilatation (laparoscopic view) with centrilobular necrosis (histologic view). **(K, L)** A case of severe liver injury with moderate steatosis and severe sinusoidal congestion.

The liver toxicity was graded by the intra-operative laparoscopic view of the liver surface. We classified the morphologic liver injury as severe: when both sinusoidal dilatation and steatosis represented more than one-third of the laparoscopic view of the liver surface; moderate: when sinusoidal dilatation occupied more than one-third but steatosis was noted for less than one-third of the liver surface, or vice versa; mild: when both steatosis dilatation and steatosis accounted for less than one-third of the liver surface. We validated the laparoscopic grading of liver toxicity based on our pilot study which showed the close correlation between laparoscopic view and histopathology (Supplementary Videos 1-7), in which presence of sinusoidal dilatation (SD) was recorded using the Rubbia-Brandt Score [27] as follows: 0, absent; 1, mild (centrilobular involvement limited to one-third of the lobular surface); 2, moderate (centrilobular involvement extending to two-thirds of the lobular surface); 3, severe (complete lobular involvement), and the liver steatosis was graded from 0 to 3: absent~5% (grade 0), 5%~33% of hepatocytes (grade 1), between 33% and 66% (grade 2), and >66% (grade 3) (Figure 4, A~F).

### Assessment of safety profiles

The adverse effects of CCRT and surgical complications of both treatment groups were well recorded in the case report forms, focusing on the bevacizumab and cetuximab-related side effects and any severe adverse events that caused the discontinuation of treatment.

### Statistics

All patients were prospectively followed up from the initial diagnosis of cancer until November, 2016. Data were assessed according to the intention-to-treat principle. Patients who died without a reported tumor recurrence were assumed to have had a recurrence at death unless it was clearly demonstrated otherwise, in which case the patients’ data were censored on the date of death in the analysis of the time-to-recurrence. Kaplan-Meier curves were constructed to estimate the distribution of the disease-free survival. The primary analysis consisted of a two-sided log-rank test comparing time with recurrence among patient groups. In evaluating secondary endpoints, two tailed Fisher's exact test or Chi-square test with or without Yates’ correction was appropriately used to analyze the categorical data, whereas continuous data were compared by Student's *t*-test. The significance level of all tests was set at p<0.05.

## SUPPLEMENTARY MATERIALS FIGURES AND TABLES

















## Abbreviations

CCRT: Concurrent chemoradiation therapy
FOLFOX: folinic acid + leucovorin + oxaliplatin
TME: Total mesorectal excision
