# Supplementary material for: Treatment outcomes regarding the addition of targeted agents in the therapeutic portfolio for stage II-III rectal cancer undergoing neoadjuvant chemoradiation
Source: Oncotarget. 2017 Oct 10;8(60):101832–46. doi: 10.18632/oncotarget.21762 (PMC5731917; doi:10.18632/oncotarget.21762)
Supplement: Supplementary file 1 [file oncotarget-08-101832-s001.pdf]

Treatment outcomes regarding the addition of targeted agents in the therapeutic portfolio for stage II-III rectal cancer undergoing neoadjuvant chemoradiation

SUPPLEMENTARY MATERIALS

Supplementary Video 1: Video of control normal liver.

See Supplementary Video 1

Supplementary Video 2: Mild steatosis and sinusoidal dilatation.

See Supplementary Video 2

Supplementary Video 3: Mild steatosis without sinusoidal dilatation.

See Supplementary Video 3

Supplementary Video 4: Moderate liver toxicity.

See Supplementary Video 4

Supplementary Video 5: Severe liver toxicity with predominant sinusoidal dilatation.

See Supplementary Video 5

Supplementary Video 6: Severe liver toxicity with predominant sinusoidal.

See Supplementary Video 6

Supplementary Video 7: Equal severity of steatosis and sinusoidal diatation.

See Supplementary Video 7
